# Stability in fecal metabolites amid a diverse gut microbiome composition: a one-month longitudinal study of variability in healthy individuals

**DOI:** 10.1080/19490976.2024.2427878

**Published:** 2024-11-12

**Authors:** Matteo Sangermani, Indri Desiati, Solveig M. Jørgensen, Jia V. Li, Trygve Andreassen, Tone F. Bathen, Guro F. Giskeødegård

**Affiliations:** a Department of Public Health and Nursing, NTNU, Trondheim, Norway; b Department of Surgery, St. Olavs University Hospital, Trondheim, Norway; c Department of Circulation and Medical Imaging, NTNU, Trondheim, Norway; d Department of Metabolism, Digestion and Reproduction, Imperial College London, London, UK; e Central Staff, St. Olavs Hospital HF, Trondheim, Norway

**Keywords:** 16S rRNA, fecal metabolites, gut microbiome, longitudinal study, multi-omics, NMR

## Abstract

An extensive network of microbial-host interactions exists in the gut, making the gut microbiome a complex ecosystem to untangle. The microbial composition and the fecal metabolites are important readouts to investigate intricate microbiota-diet-host interplay. However, this ecosystem is dynamic, and it is of interest to understand the degree and timescale of changes occurring in the gut microbiota, during disease as well as in healthy individuals. Cross-sectional study design is often used to investigate the microbiome, but this design provides a static snapshot and cannot provide evidence on the dynamic nature of the gut microbiome. Longitudinal studies are better suited to extrapolate causation in a study or assess changes over time. This study investigates longitudinal change in the gut microbiome and fecal metabolites in 14 healthy individuals with weekly sampling over a period of one-month (four time points), to elucidate the temporal changes occurring in the gut microbiome composition and fecal metabolites. Utilizing 16S rRNA amplicon sequencing for microbiome analysis and NMR spectroscopy for fecal metabolite characterization, we assessed the stability of these two types of measurable parameters in fecal samples during the period of one month. Our results show that the gut microbiome display large variations between healthy individuals, but relatively lower within-individual variations, which makes it possible to uniquely identify individuals. The fecal metabolites showed higher stability over time compared to the microbiome and exhibited consistently smaller variations both within and between individuals. This relative higher stability of the fecal metabolites suggests a balanced, consistent output even amid individual’s differences in microbial composition and they can provide a viable complementary readout to better understand the microbiome activity.

## Introduction

The gastrointestinal tract is known to host a diverse ecosystem of microorganisms contributing to a wide range of metabolic processes. The large metabolic potential of this diverse community makes it capable to break-down and release many compounds via an extensive cross-feeding network that feeds both the microbiota and its host. Metabolites produced by certain microbes are used as precursors by other microbes, which in turn can feed on those or create different metabolites. In mice it has been shown that different gut microbiota are capable of inducing changes in host’s dietary choices, reflecting the unique metabolic requirements of each microbial community.^1^ This in turn result in a different array of metabolic compounds circulating and nourishing the host. Similarly, the by-product of the microbial metabolic activity can create metabolites that have wide range systemic effects on the health of the human host.^2^

The gut microbiome is important for the host as source of micronutrients, molecular precursors and other building blocks, such as carboxylic acids.^3^ Undigested peptides that reach the colon provide crucial precursors for the synthesis of, e.g., signaling molecule, indoles, hormones, and short chain fatty acids (SCFAs).^4^ Past studies have established a direct link between a protein-rich diet and the formation of a gut environment producing microbial-derived metabolites detrimental to the host, e.g., in the case of inflammatory bowel disease (IBD) and colorectal cancer.^5,6^ SCFAs are derived mainly from the fermentation of carbohydrate, most commonly from dietary fibers, but SCFAs can also be produced by fermentation of amino acids.^7^ SCFAs play a pivotal role in the well-being of the intestinal epithelial cells, which in turn can produce mucus and maintain a healthy homeostasis in the gut ecosystem.^8,9^ SCFAs can also regulate the immune system, T-cells maturation, and inflammation state at the gut mucosa.^10,11^

Most studies on the human microbiome are cross-sectional studies, only sampling one time point per individual. However, longitudinal studies of the microbiome provide advantages in understanding the dynamic nature of the microbiome as they assess changes over time and may inform on causation between the gut microbiome and health. It is well established that differences in diet can explain some of the heterogeneity in microbial composition of a population.^12,13^ However, many factors can alter the composition of the microbiome over long periods of time, for example, development during infancy, aging and the use of antibiotics.^14–16^ A few studies have explored the short time changes in the microbiome of healthy individuals. A study sampling a cohort of individuals daily for about 2 weeks found that dietary diversity was associated with higher stability in the microbiome composition.^17^ Moreover, when comparing people with omnivore diet to those with a plant-based diet, the inter-individual variation of the microbiome remained high, even between people consuming the same diet, but it resulted in the production of diet-dependent serum metabolites.^18^ A study that characterized the microbiome composition and gene expression (i.e., metatranscriptomics) suggested that due to its stability the composition is more suited to inform on long-term effects, whereas metatranscriptomics will reflect small perturbation in microbial activity and is suited to investigate short-term correlations.^19^

The fecal metabolome are the culmination of metabolic pathways and microbial-host interactions. Fecal metabolites can serve as biomarkers that reflect the complex interplay between the gut microbiota, diet, and host physiology. A study in twins showed that around two thirds of the variance in the fecal metabolome can be ascribed to the microbial composition, and interestingly, only a minor portion of the variance is due to the host genetics.^20^ A recent study analyzing both blood serum and fecal metabolites showed that the latter were better associated with several cardiometabolic diseases and provided better predictors.^21^ However, there is a gap in knowledge regarding the magnitude of changes that occur in the fecal metabolites over time, and how changes in the fecal metabolite correlates to the microbiome composition, specifically over short-time periods.

There is considerable degree of uncertainty on how often to sample individuals during a clinical study. Planning must balance the advantage of having additional time points, experimental costs, as well as the expected rate of change in health, e.g., caused by the disease under investigation. However, without having a clear picture of the rate of changes in the microbiome of healthy individual, it is difficult to predict when one may incur in over- or under-sampling. To address this lack of knowledge, we investigated changes in the microbiome composition and fecal metabolites in healthy individuals over a period of one month. In this study we describe the stability of the gut microbiome and its metabolites, and we assessed the changes in the gut microbiome composition associated with changes in fecal metabolite.

## Material and methods

### Cohort and sample collection

We recruited a cohort of 14 volunteers (A01 to A14) that provided fecal samples at four different time points for one month: at day 0, day 7, day 14, and day 28. Participants were to the best of our knowledge healthy, did not report any intestinal problems or disorders. Moreover, we included only participants that had no antibiotics treatment both in the 6 months prior, and excluded those that underwent antibiotics treatment or took other medicaments during the sampling period. Individuals were instructed to keep their usual diet and behavior for the entire period of the study. We only included one participant per family or cohabitation units, and all participants lived in the city of Trondheim, Norway. The average age of the cohort was 37.0 ± 8.4 years (34.2 ± 4.5 years for males; 39.1 ± 10.2 years for males; mean ± SD). Six individuals we male, and eight individuals were female. The average BMI of the cohort was 23.6 ± 2.2 (23.1 ± 1.5 for males; 23.9 ± 2.6 for females).

Sample collection was performed by the individuals at home, using the following procedure: fecal samples were collected using the Fe-Col fecal catcher (Fe-Col, cat: FC2010, AlphaLaboratories, UK) in the toilette seat and a subsample was transferred into a sterile 15 mL cryotube. Fecal material was immediately stored at ~ 4°C in home refrigerator and delivered for storage within 24 hours, where they were stored at −80°C until further analyses. Participants’ age, sex and medication use were recorded at each sampling, and samples were anonymized. Lastly, to assess the reproducibility of methods and to compare longitudinal variability of samples, quality control samples (*n* ≥ 3) were prepared from fecal samples of a single volunteer (not included in the study cohort).

### DNA sequencing, library construction and sequencing

DNA extraction from fecal samples was carried out for the entire cohort in a randomized fashion. Fecal samples were thawed for about 20 min at room temperature, to allow samples to soften before taking a subsample. Around 170–200 mg of fecal material was then processed using QIAamp PowerFecal Pro DNA Kit (cat: 12830–50, Qiagen). The template DNA extracted was eluted using nuclease-free water (W4502, Sigma-Aldrich), and stored in low binding Eppendorf tubes (0030108051, Eppendorf) at −20°C. DNA samples were quantified by Qubit HS-DNA assay (Thermo Fisher), and samples with < 100 ng DNA were reextracted. Libraries were created with extracted DNA as templates, using the QIAseq 16S/ITS Region Panel kit (333842, Qiagen). The kit allows to target and amplify multiple regions of the 16S rRNA gene at the same time. We targeted the amplification of the regions V1V2 and V5V7 (target areas of primers are approximately 350 nt and 410 nt, respectively). We multiplexed to sequence 96 samples in one batch, using QIAseq 16S/ITS Index kit, set A (333822, Qiagen). Sequencing pair-ended (2× 300 bp) was performed on a MiSeq V3 flowcell platform (Illumina). Combined with the QIAseq 16S/ITS Region Panel kit, which uses phased primers, we generally obtained outputs of around 20 M raw after filtering PhiX spacer reads. Sequencing was performed at the Genomics Core Facility, NTNU.

### Bioinformatics for sequencing data

A pipeline was created in QIIME2 to analyze the raw sequencing and yield taxonomic features of the gut microbiome.^22^ We screened for potential human contaminants with Bowtie2 v.2.3.036 and employed DADA2 as denoising algorithm to merge and quality filter sequences.^23^ ASVs were assigned a taxonomy using SILVA (release 132). The main parameters for the denoise algorithm were the following: no trimming (–p-trim-left-f and -r set at 0), low-quality bases were truncated at 240 nt for forward reads (–p-trunc-len-f 240) and at 200 nt for reverse (–p-trunc-len-r 200), and we set maximum expected errors for forward reads at 6 (–p-max-ee-f 6) and at 8 for reverse (–p-max-ee-r 8). The taxonomic features from the two individual regions (V1V2 and V5V7) were used as a combined dataset. On average 20–22 million raw reads were generated per sample. On average 95% of the reads passed decontamination and quality-trimming steps for downstream analysis, and the rest were discarded from further analysis. We performed a rarefaction graph to ensure that sequencing was done with sufficient depth. We had on average 125 000 reads per sample and reached a plateau in the rarefaction curve at around 30 000 reads. Sequencing quality of sample A12 on day 28 was low, with less than 1000 reads, and was excluded from further analysis. ASVs (in total 5327 for the entire cohort) were filtered and we removed those that had a relative abundance of less than 0.03% in more than 10% of all samples. After filtering, the data set consisted of 3470 unique ASVs as taxonomic features of the entire cohort, which accounted for a total of 148 genera, from 53 families, and 16 classes (Suppl. Table S1).

### Fecal metabolites extraction

Our method for the extraction of aqueous phase metabolites of fecal samples was developed by optimizing and combining previously published studies.^24,25^ In detail, fecal samples were thawed for about 20 min at room temperature. Subsequently, fecal material (~750 mg) was transferred into sterile Eppendorf tubes (5 mL) and mixed with extraction buffer (1.5 mL, creating a 1:2 ratio). The extraction buffer consisted of 0.25 mm NaN_3_ in PBS (with PBS formulation being: 8.00 g/L NaCl, 0.20 g/L KCl, 1.4 g/L Na_2_HPO4 and 0.24 g KH_2_PO4). All samples were vortexed at the highest speed in a multitube vortex (5 min), and subsequently placed in a ultrasonication bath (20 min). The liquified mixture of fecal sample and extraction buffer was centrifuged (30 min at 10,000 × g) at 4°C in a benchtop centrifuge with swinging bucket rotor. After centrifugation, a least 1 mL of the supernatant was collected from the samples and transferred to clean 1.5 mL Eppendorf tubes. Sample extractions were carried in a randomized fashion and tubes were stored at −20°C until NMR measurements, which were conducted as a single batch.

### NMR measurements

Metabolomic characterization was done by nuclear magnetic resonance (NMR) spectroscopy, using a modified version of a protocol used in previous studies.^26^ For NMR measurements, 630 µL of the extracted metabolites were mixed with 70 µL NMR buffer (1:10 ratio) and mixed by vortexing at highest speed for a few seconds. The NMR buffer contained deuterium oxide (D_2_O) with the following composition: 1.5 M KH_2_PO_4_, 2 mm NaN_3_ and 0.1% TSP (pH adjusted to ~ 7.4). Using sterile glass pipettes, the mixture was transferred into 5 mm NMR tubes, filling up to a volume of about 600 µL (~4 cm from bottom of the tube). The NMR tubes were placed in the NMR instrument with autosampler for analysis and measured within 12 hours (storage at 6°C).

1D ^1^H NOESY spectra of all samples were obtained using a 600 MHz Bruker Avance NEO NMR spectrometer equipped with a BBI probe and automation via SampleJet (Bruker Biospin GmbH, Rheinstetten). We used the pulse program “noesygppr1d” (1D NOESY with water pre-saturation during relaxation delay and mixing time, and spoil gradients). The acquisition parameters were: acquisition time 2.75 s, spectral width 19.83 ppm, number of data points (TD) 64k and 4 s relaxation delay. The number of scans were 32 and 4 dummy scans, with a total experiment time per spectra of 4 min and 7 s. During the acquisition, the temperature was kept stable at 300 K. FIDs were processed with exponential line broadening of 0.3 hz and zero filled to 128k data points prior to Fourier transformation.

### Quantification of metabolites

Chenomx NMR Suite 8.6 Standard version and 9.0 Pro version (Chenomx Inc., Edmonton, AB, Canada) was used to identify and quantify fecal metabolites. For each spectrum, the baseline was corrected automatically using the Whitaker cubic spline function with minor manual adjustments. Due to differences in water content of fecal samples, we performed relative quantification, whereby the metabolite levels were normalized to the total metabolite level for each sample. We identified 102 compounds and quantified the peaks using Chenomx NMR Suite and HMDB (Human Metabolome Database) libraries. We removed 33 metabolites from further analysis, based on quality of fitting and passing a cutoff threshold of the match factor set at > 75%. The remaining 69 metabolites were used in the analysis (Suppl. Table S2).

### Data analysis

We employed the R packages tidyverse (1.3.2), dplyr (1.0.10), stringr (1.5.0), and mixOmicx (6.24.0) for data handling, and results were visualized using ggplot2 (3.4.0). We calculated the Shannon index for the alpha diversity (measuring within-sample diversity) and Jaccard index for the beta diversity (measuring differences in diversity between samples). In both cases we used genus level as input features. The metric Jaccard index was calculated by pairing day 0 to either day 7, 14 or 28. The coefficient of variation (CoV) was calculated to assess the stability of individual features over time, for individual taxonomic features and metabolites. The t-distributed stochastic neighbor embedding (tSNE) algorithm was used to visualize the within- and between-individual variation in the microbiome composition and fecal metabolites (R package Rtsne (0.16)). The data was mean centered and scaled before performing tSNE with perplex parameter set to 5, and we used genus level as input features for the algorithm. Partial Spearman’s Rank Correlation between metabolites and microbiome was calculated using R package PResiduals (1.0–1) and plotted with corrplot (0.92) to evaluate the associations between the microbiome features and the fecal metabolites. The Partial Spearman’s Rank Correlation was run adjusted for sex, age, and individual.

## Results

### Longitudinal characteristics of the gut microbial composition

**Figure 1.**
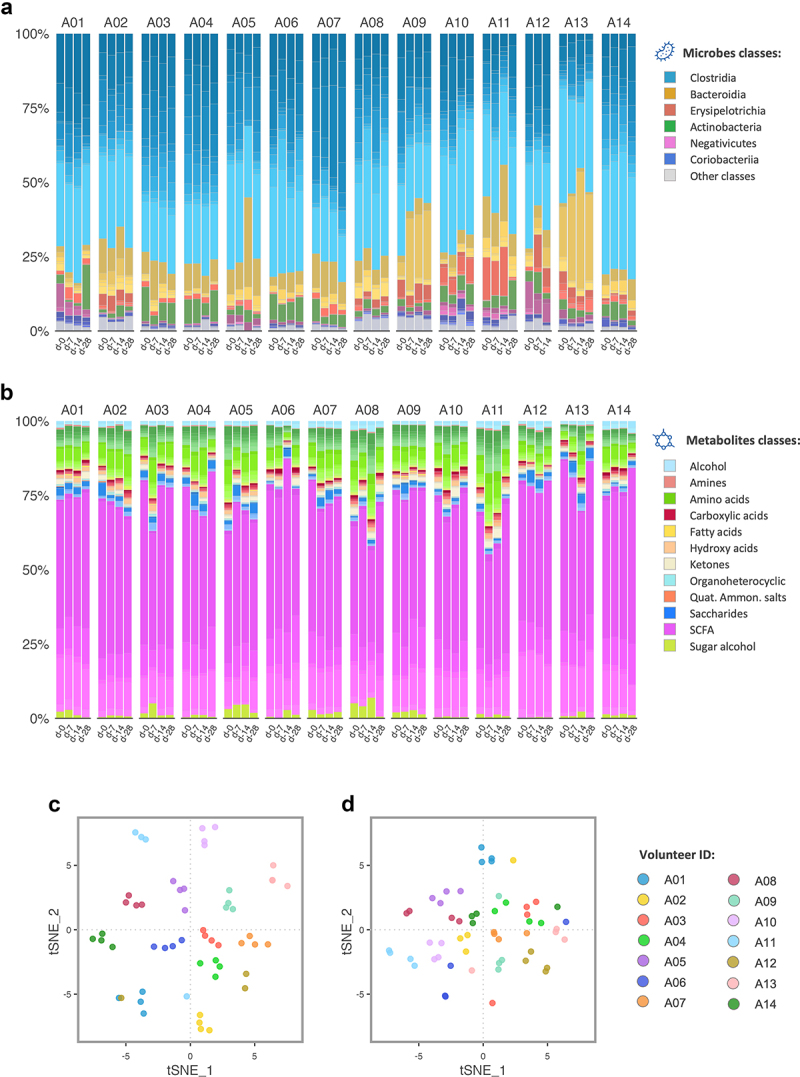
Composition of fecal microbiome and metabolites in the study cohort.

Figure 1 shows the composition fecal microbiota (Figure 1a) and the fecal metabolites (Figure 1b). over the four sampling time points (day 0, 7, 14 and 28). Each individual displayed a microbiome composition that visually appears relatively similar throughout the sampling period of one month (Figure 1a,c). Dimensional reduction via tSNE analysis shows that samples from the same individual tend to cluster together based on the microbiome profile, whereby individuals are well separated from one another (Figure 1c).

a and b) The composition of the gut microbiome (a) and fecal metabolites (b) over the course of a month. The figures show data for the cohort of 14 volunteers (A01 to A14) that provided fecal samples at 4 time points (day 0, 7, 14 and 28). Each stacked bar plot column displays the relative abundance of the features characterizing a sample: microbial genera (in a), and measured metabolites (in b). In (a), genera from the same taxonomic class share the same colour, but are visualized by different colour saturations. Moreover, classes with average abundances < 0.3% were merged into one common block, named “Other classes”. Similarly, in (b) metabolites belonging to the same class are filled with a graded range of the same colour. c and d) Multidimensional reduction via tSNE algorithm. Two latent variables are plotted to show clustering of samples analysed using the microbiome (c) and fecal metabolomics data (d). The samples are coloured by individual.

The classes Clostridia, Bacteroidia, Actinobacteria and Erysipelotrychia were present in all individual’s samples with an average relative abundance of 70.1%, 13.9%, 6.3% and 4.3%, respectively. Together these classes account for about 95% of all microbes in the gut ecosystem. The Clostridia (Figure 1a) was by far the dominant class within the gut microbiome of all individuals. The abundance of the remaining classes was relatively constant within one individual but varied between individuals. For example, individual A01 had both Negativicutes and Coriobacteria at high levels, whereas levels of Bacteroidia and Actinobacteria were low. On the other hand, individual A02 had higher abundance of Bacteroidia, followed by Erysipelotrychia, and very low amounts of Negativicutes and Coriobacteria. The most frequent genera were *Blautia*, *Faecalibacterium*, *Bacteroides*, *Bifidosbacterium*, *Anaerostipes* and *Agathobacter*; each of which on average account for > 5% of the composition and belong to the class Clostridia, except for the genus *Bacteroides*, which belongs to the class Bacteroidia, and genus *Bifidosbacterium*, which belongs to the class Actinobacteria.

The Shannon metric was used to calculate the alpha diversity in the gut microbiome. The value distribution (ranging between 3.0 and 3.5) was comparable between the individuals (Suppl. Figure S1). The distribution of values of the beta diversity metrics (Jaccard similarity) was similar between the majority of individuals in the cohort, except for individuals A05 and A11, which show changes at day 14 and day 28, respectively, resulting in lower values. Moreover, for individual A12 the Jaccard similarity values were low, which attests to substantial changes at day 7 and 14, in respect to day 0.

### Longitudinal characteristics in the fecal metabolome

The composition of fecal metabolites was dominated by the SCFA (Figure 1b) which represented about 70% of all the metabolites measured. They are followed by assorted amino acids (Figure 1b), which all together represent about 15% of the compounds measured. Most measured compounds accounted for between 0.1% and 2% of the metabolite composition, except for the acetate, propionate, and butyrate (all SCFAs), which were by far the most abundant, constituting on average 46.7%, 11.7% and 9.4%, respectively. Within individuals, we observed only small changes in metabolites composition throughout the course of the sampling period and the composition of each individual appears less unique than the microbial composition. tSNE shows that samples belonging to an individual are partly clustered (Figure 1d), but individuals are not well separated and, in some cases, overlap (e.g., individuals A03 and A04) or spread over a large area (e.g., individual A06).

### Variability in the gut microbiome and metabolome

**Figure 2.**
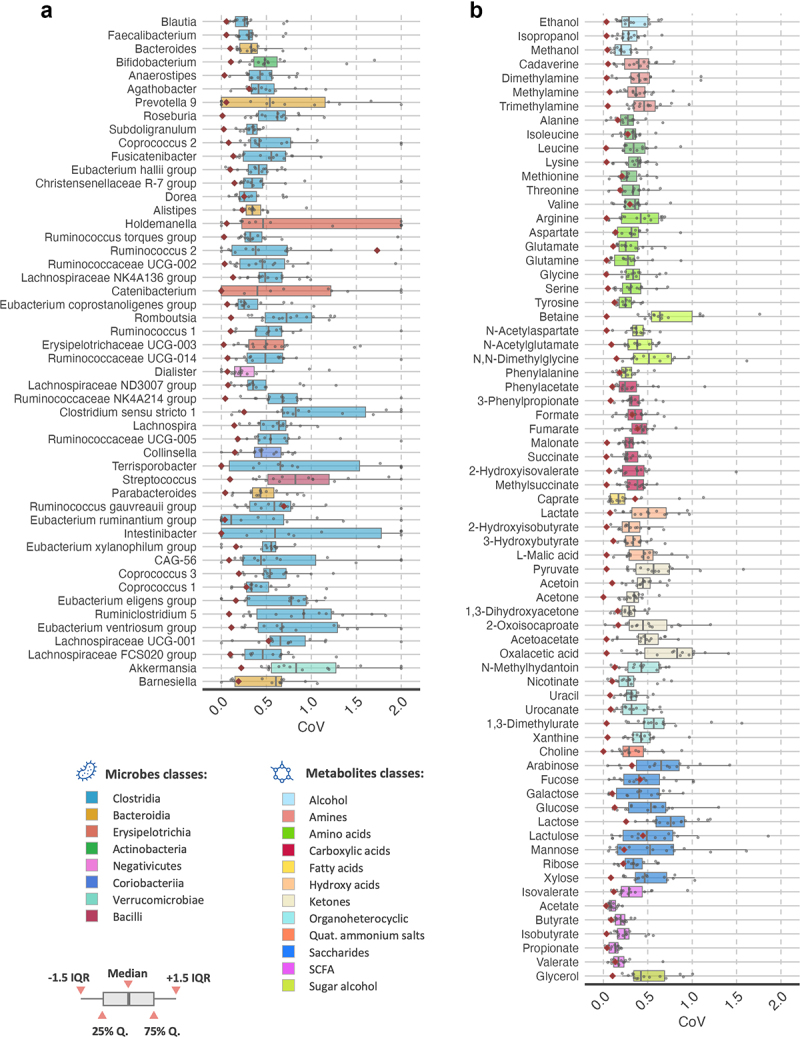
Coefficient of variation in microbial and metabolites features.

To investigate the stability in the microbiome and fecal metabolome over a month, we calculated the coefficient of variation (CoV) for each individual based on the four sampling time points. We also calculated the CoV for quality control samples as a measure of method variability (Figure 2). For the quality control samples, the genera had an average median CoV of 10.7%. The median CoV of our longitudinal cohort during one month was substantially higher than the quality control samples for the gut microbiome (Figure 2a). The genera with the highest abundance, those with relative abundance > 0.3% (in total 50 genera that account for ~ 90% of the microbiome composition), had an average median CoV of 49.3 (±17.6) % (range: 10.9% to 91.6%), whereas genera with relative abundance < 0.3% (accounting for ~ 10% of the microbiome composition) had lower average median CoV, at 22.1 (±32.5) % (range: 0.0% to 144.4%). Note that in this latter group 60 out of the 98 features had average median CoV of zero, because these less abundant genera occured only in few individuals. Considering all microbiome features (148 genera) the average median CoV was 31.2 (±31.1) %. This was similar when analyzing the CoV of families and classes (Suppl. Figure S2). The most abundant class, Clostridia, had a median CoV of 5.7%; while the next most abundant classes, Erysipelotrichia and Negativicutes (both of the phyla Firmicutes), class Bacteroidia and Actinobacteria, had median CoV ranging between 23.7% and 48.2% (Suppl. Figure S2). The interclass correlation (ICC) values were on average 0.51 for the top 50 genera (accounting for ~ 90% of the microbiome composition), and 0.40 when including all genera (Suppl. Figure S3).

a and b) In (a) the chart shows the coefficient of variation (CoV) for the genera of the gut microbiome. Genera are sorted by abundance and only the top 50 most abundant genera are displayed. (b) Shows the CoV of the fecal metabolites. In the charts the red dots are control values, CoV calculated from set of quality control samples (*n*≥3). Boxes are coloured according to the features hierarchical grouping (see legend). Box plot – line, mean; box, delimits 25^th^ and 75^th^ percentile; whiskers, 1.5 interquartile; points, individual values.

For fecal metabolites, the CoV of the quality control samples had an average median value of 11.5% (Figure 2b). It is worth noting that the average median CoV of metabolites for the longitudinal cohort was 37.0 (±13.4) %, which is significantly lower than that of the top 50 microbial genera (t-test, p < 0.05). SCFAs had the lowest median CoV, with an average of 18.5 (±7.2) %. Saccharides and ketones had the highest average median CoV, respectively at 50.7 (±13.1) % and 48.5 ± (17.6) %, Acetate, Butyrate, Propionate and Valerate, all SCFAs, had the lowest average median CoV values at 8.6%, 19.5%, 13.3% and 17.0%, respectively. The CoV of amino acids (essential and non-essential) was around (31.7 ± 5.1) %, whereas amino acids derivates (e.g., Betaine and N,N-dimethylglycine) had average median CoV, at 43.1 (±14.8) %. Moreover, the IQR of the distribution of CoV values were smaller for metabolites than for microbiome features, indicating within cohort variability was smaller for the metabolites data. Plotting the distribution of CoV by individual showed the same outcome (Suppl. Figure 4S), namely that metabolites possess smaller median CoV values and shorter IQR than taxonomic features. With few exceptions, the taxa or metabolites that have the highest relative abundance showed the lowest median CoV and IQR ranges (Suppl. Figure S5). The IQR for microbiome features was 49.1 (±40.4) % (average IQR ± sd), whereas for metabolites it was 24.0 ± (12.7) %. In some classes of metabolites, such as SCFAs and amino acids, the IQR was even lower, at 12.7 (±5.7) % and 21.7 (±10.6) %, respectively. The average ICC of metabolites was 0.40 (Suppl. Figure S3).

### Associations between gut microbial composition and metabolites

**Figure 3.**
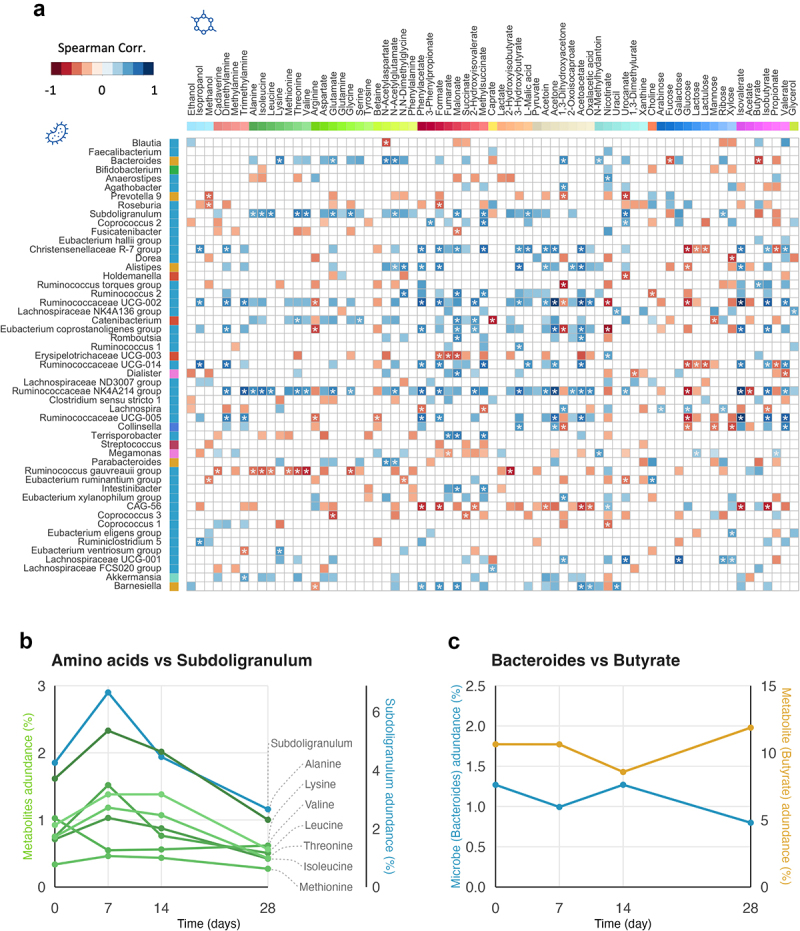
Correlation between genera and metabolites and representative example of time changes.

To investigate the association between gut microbiota and fecal metabolites, we calculated the Spearman correlation between microbes and fecal metabolites using data from all individuals and time points. Our results show that some significant and strong correlations exist between individual metabolites and specific taxa. However, no clear pattern of correlations emerged concerning specific taxa and classes of metabolites. The genera *Bacteroides*, *Subdoligranum*, *Alistipes*, and several *Ruminococcous* taxa had the greatest frequency of significant correlations, with between 10–25% of fecal metabolites (p < 0.05, adjusted with Benjamin-Hochberg) (Figure 3a). The families Ruminococcaceae, Christellaceae, and Familiy XIII, all three belonging to the class Clostridia, had significant correlations with 25–30% metabolites (p < 0.05, adjusted with Benjamin-Hochberg). The three most abundant families, Lachnospiraceae, Bacteroidaceae and, Rumicoccaceae (Suppl. Table S1 and Suppl. Figure S2), had significant correlations (ranging between 12–25% of metabolites) especially within the metabolite belonging to the classes amino acids, SCFAs, ketones and carboxylic acids (Suppl. Figure S6B, 6C, and 6D). On the other hand, taxa belonging to the families Bifidobacteriaceae, Erysipelotrichaceae and Prevotellacea had almost no significant correlations with metabolites. The metabolites malonate, phenylacetate, formate, 1.3-Dihydroxyacetone and acetoacetate had the highest number of significant correlations, with at least 20% of the top 50 most abundant bacterial genera. Amino acids, carboxylic acids and SCFAs were the classes of metabolites with the most correlations to the gut microbiota taxa (Figure 3a and Suppl. Figure S6). The genera *Bacteroides* and *Subdoligranulum* showed correlations to several essential amino acids, e.g., alanine, leucine, lysine, threonine, valine, glutamate, and N-acetylglutamate (adjusted p < 0.05; Figure 3a). This can be followed over time, by observing the changes in essential amino acids levels in relation to the relative abundance of the genus *Subdoligranulum* or the family Bacteroidaceae (Figure 3b and Suppl. Figure S6b, respectively; both showing representative individuals). Genus Bacteroides also shows strong negative correlation with the metabolite butyrate, and time evolution reflects this as well (Figure 3a,c).

a) Spearman correlation between the taxonomic features (genera; y-axis) and metabolites (x-axis) in fecal samples, considering all time points. Top 50 genera are displayed in order of relative abundance. Only significant correlations (with nominal *p* < .05) are displayed with a colour range proportional to the correlation values (Red for negative correlation and blue for positive correlation). A star symbol is placed inside squares to mark correlations where *p* < .05 after Benjamin-Hochberg adjustment. The coloured squares below the labels for the metabolites (top x-axis) indicate the metabolites classes and squares next to the genera name indicate microbial classes (see legends Figure 2a and 2b for reference). b) Temporal changes in abundance of genus *Subdoligranulum* and essential amino acids in a representative individual. c) Changes in abundance of genus *Bacteroides* and metabolite butyrate in a representative individual.

## Discussion

This study explored the longitudinal variations in the gut microbiome composition and fecal metabolites over a one-month period in a cohort of healthy individuals. This study is unique in exploring the correlation between microbiome composition and metabolites in a short-scale longitudinal study of a cohort of healthy individuals. The longitudinal variation was generally higher than for the repeated quality control samples, both for gut microbiome composition and fecal metabolites. This indicates that the underlying biological variability – such as due to daily changes in food intake and other lifestyle factors – is consistently greater than methodological variability. Despite this, the composition of the gut microbiome can be considered relatively stable over the course of one month because the variability between individuals is greater that the within-individual variation and allows to identify distinctive microbial profiles of healthy subjects. This is in line with the view of the microbiome as composed of a stable core which is persistent and resilient to changes, and a flexible set of microbes that can more readily respond to changes.^27^

The most abundant classes, Clostridia and Bacteroidia, form a core set of microbiota that remains relatively constant, in line with past studies investigating the microbiome composition in western countries.^19,28^ Low abundance taxonomic features had higher median CoV, likely because they are present at highly dissimilar amounts in each individual, which suggest that taxa with higher variability constitute part of the flexible microbiome. These more flexible components of the microbiome accounts for a large between-individual variations, but a relatively smaller within-individual variations. This in turn allows the microbiome to be used as a sort of fingerprint that can uniquely identify individuals.^29,30^

The narrow distribution of Shannon index values indicates that all participants had comparable alpha diversity in their gut microbiota. Also, no large variations in alpha diversity were observed during the one-month study period, as expected for a cohort of healthy volunteer that had no systematic lifestyle changes, except for single time points in two of the individuals (for example, A11 and A12). In such cases we observed in the microbiome dataset (Figure 1c) that one timepoint was set far apart from the other timepoints. Individual A12 coincidently had a low Jaccard similarity index, indicating a less stable microbiome composition. On the other hand, individuals A01 and A05 had an outlier time point each (Figure 1c) that clustered apart, but to a lesser degree. These observations can be explained by the degree of short-term changes occurring in microbial composition of each individual, the larger the changes, the larger the spread of outlier points. Moreover, using individuals A01, A11 and A12 as examples, despite outlier timepoints in the microbiome dataset, these individuals clustered much closer when analyzing the metabolite dataset. This indicates that changes in microbiome composition do not result in large shifts in metabolic profile, reflecting a maintenance of overall baseline microbial activity.

Fecal metabolites are the result of the microbiome-host interaction, and as such are the product of gene expression levels in the microbiota and host. Previous studies have shown that over short term, metatranscriptomics has lower stability than compositional data, reflecting dynamic changes in gene expression occurring faster than compositional changes,^19^ an expression of the high turnover of the mRNA in microbial cells.^31^ In fact, adequate and immediate stabilization of fecal sample right after defecation is necessary to preserve high quality mRNA and attempt the delicate extraction process.^32^ Since the microbial community is alive, long sampling time or defreezing could result in rapid changes in the gene expression of the microbial community. This would imply that the fecal metabolome should be quite changeable since it reflects more closely changes in gene expression. The ICC of metabolites and microbial genera was comparable. On the other hand the CoV was larger on average for microbial genera than metabolites, indicating moderately better stability of metabolites over microbial genera. These suggest that fecal metabolites were more stable over the course of one month than taxonomic features. Despite larger stability, fecal metabolites had smaller inter-individual variability than the composition.

The longitudinal stability of the fecal metabolome observed may appear counter intuitive. However, the higher variability of the gut microbiome composition does not necessarily equate in functional changes of the community, and in fact, the gut ecosystem has been shown to display a notable degree of robustness in its metabolic functions even amidst shift in composition.^19,27^ Previous studies have observed short-term stability in the microbiome composition^17^ and its higher influence in determining the fecal metabolome over other factors, such as host genetics.^20^ Moreover, there is substantial functional redundancy in the gut microbiota, whereby diverse taxonomic groups contribute to a diverse array of metabolic pathways and ensure functional resilience.^33,34^ Together with our finding, the short-term stability of the gut microbiome composition suggests an even greater stability of the fecal metabolome due to consistent functions maintained by the gut ecosystem in healthy individuals.

The fecal metabolome composition was dominated by short-chain fatty acids (SCFAs), followed by assorted amino acids. SCFAs butyrate and acetate alone represented a large proportion of the metabolites measured. This observation is consistent with previous studies, highlighting the pivotal role SCFAs have in maintaining intestinal homeostasis and their role in the bidirectional communication between the gut microbiota and the host.^35^ Moreover, the temporal alignment between the amino acid levels and relative abundance of *Bacteroides* confirms the established role that this genus has in amino acid metabolism(. Additionally, we found correlations between butyrate-producing bacteria genus *Roseburia* and *Bacteroides* with SCFAs.^35^ Our results show specific microbiome-metabolite correlations, rather than systematic correlation between groups of compounds and large number of bacterial species. This reflect a larger inter-individual variability, but it is also a possible demonstration of functional redundancy and of how diverse groups of microbes contribute to pathways based on the ecosystem in which they are set; i.e., that composition of other symbiotic and antagonistic microbes can coordinate the phenotypic behavior.

Some important microbial metabolites were not quantifiable in our study, such as tryptophan or TMA. Tryptophan is the precursor to a large number of microbial synthesized compounds, such as nicotinate and serotonin neurotransmitters,^36^ whereas TMA is released by microbes upon digesting choline, which is mostly supplied by animal rich diets.^37^ Several other compounds overlapped in the same spectra area where TMA is located, making it impossible to univocally identify the TMA peak. Similarly, most carbohydrates reside in a dense area of the spectra and very few could be confidently identified. A 2D NMR acquisition (e.g.,^1^H-^13^C Heteronuclear Single Quantum Coherence (HSQC) spectroscopy) would be necessary to confirm the identities of peaks that are too close to one another in the 1D ^1^H spectra. In our study we opted for a water-based extraction, thus, we mostly detected apolar or slightly polar compounds that are hydrophilic. Performing extraction using an organic solvent (e.g. methanol or chloroform:methanol:H_2_O) would efficiently extract such compounds, like longer chain fatty acids and bile-derived compounds. For example, we detected two bile acids (cholate and glycocholate) in our samples, but the requirements for quality of fitting and match factor threshold were not met for inclusion in the final analysis. We removed 33 metabolites from further analysis, based on quality of fitting and passing a cutoff threshold of the match factor set at > 75%. The remaining 69 metabolites were used in the analysis

Our study cohort was of homogenous age, BMI and equal distribution of genders; thus, we detected consistent results, despite the small study size. This can, however, present some limitations since the cohort is composed of people from a restricted location and ethnicity (mostly Nordic Europe), and to date we are aware of substantial differences, for example, within the microbiome of people from different cultures, climates, or between those living in urban and rural areas.^38^

NMR-based fecal metabolites have been shown to be capable to distinguish individuals afflicted by intestinal bowel disease from healthy individuals, mainly characterized by a lower abundance of SCFAs and increased amounts of amino acids. However, alone, the fecal metabolites become less robust to distinguish between specific intestinal diseases, such as Chron’s disease and ulcerative colitis.^39,40^ Combining the microbiome and fecal metabolites data in a multi-omic analysis was shown to have higher predictive accuracy and explaining underlying differences in metabolic pathways of individual suffering inflammatory bowel disease.^40,41^ The longitudinal stability of fecal metabolites in healthy individuals may provide lower ability to discriminate individuals in cross-sectional studies, but in long-term studies such higher stability can reduce noise and improve accuracy.

In conclusion, the study contributes to our understanding of the variability of the gut microbiome and fecal metabolites in healthy individuals over time, and how the two are associated. Fecal metabolites offer a valuable complementary readout for investigations that follows gut microbiome changes over long periods of time.

## Supplementary Material

Supplemental Material

## Data Availability

Feature tables and R scripts used for analysis have been deposited and are available at the following link: (https://github.com/sanger-matteo/Stability_fecal_metabolites_microbiome.git.)
