# Supplementary figures and images for: Stability in fecal metabolites amid a diverse gut microbiome composition: a one-month longitudinal study of variability in healthy individuals
Source: Gut Microbes. 2024 Nov 12;16(1):2427878. doi: 10.1080/19490976.2024.2427878 (PMC11562901; doi:10.1080/19490976.2024.2427878)

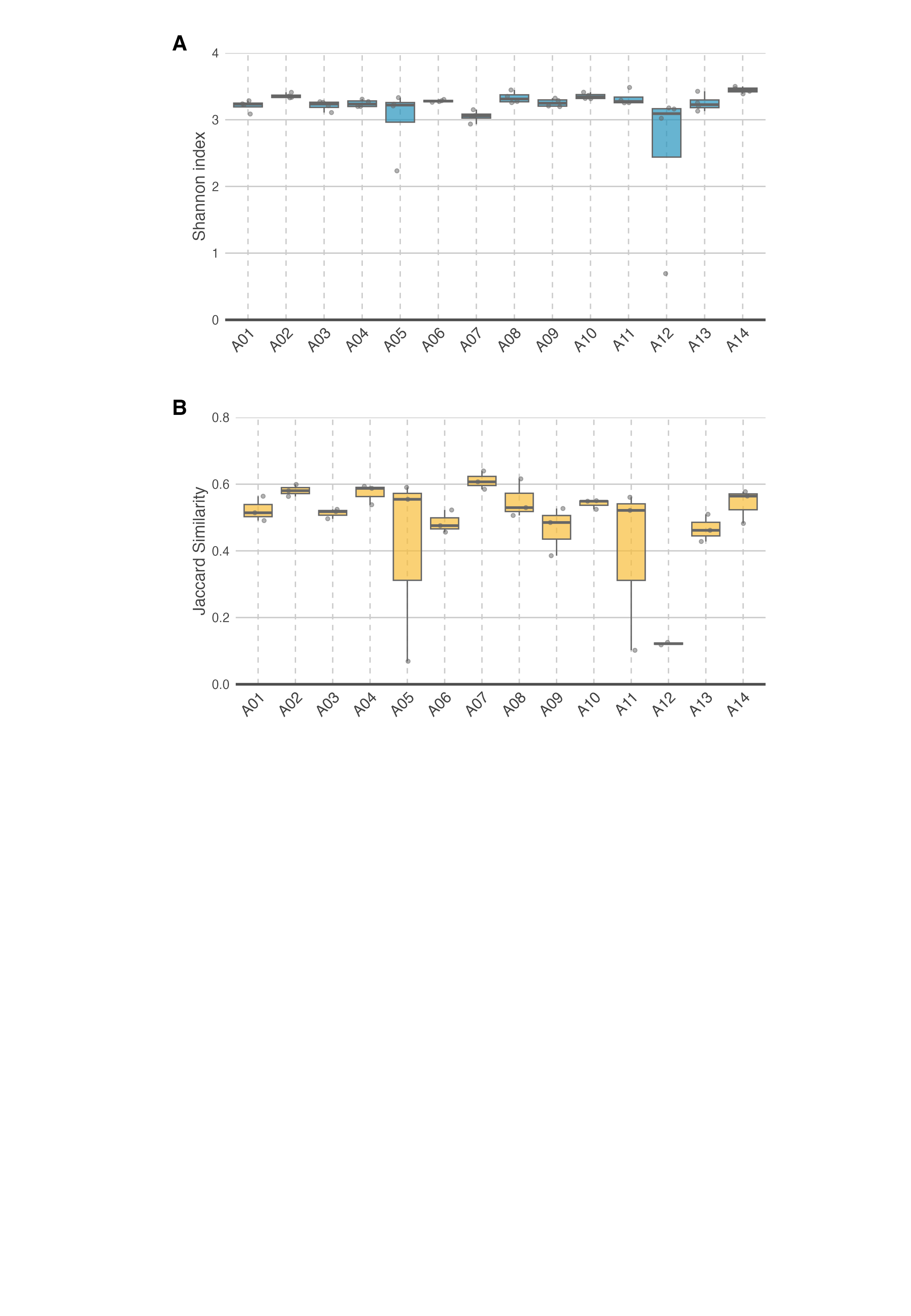

Supplement: Supplemental Material [file KGMI_A_2427878_SM0472.zip › KGMI 2427878 updated/kgmi-s-2024-0526-20241106211645/Suppl_Figure_1.tiff]

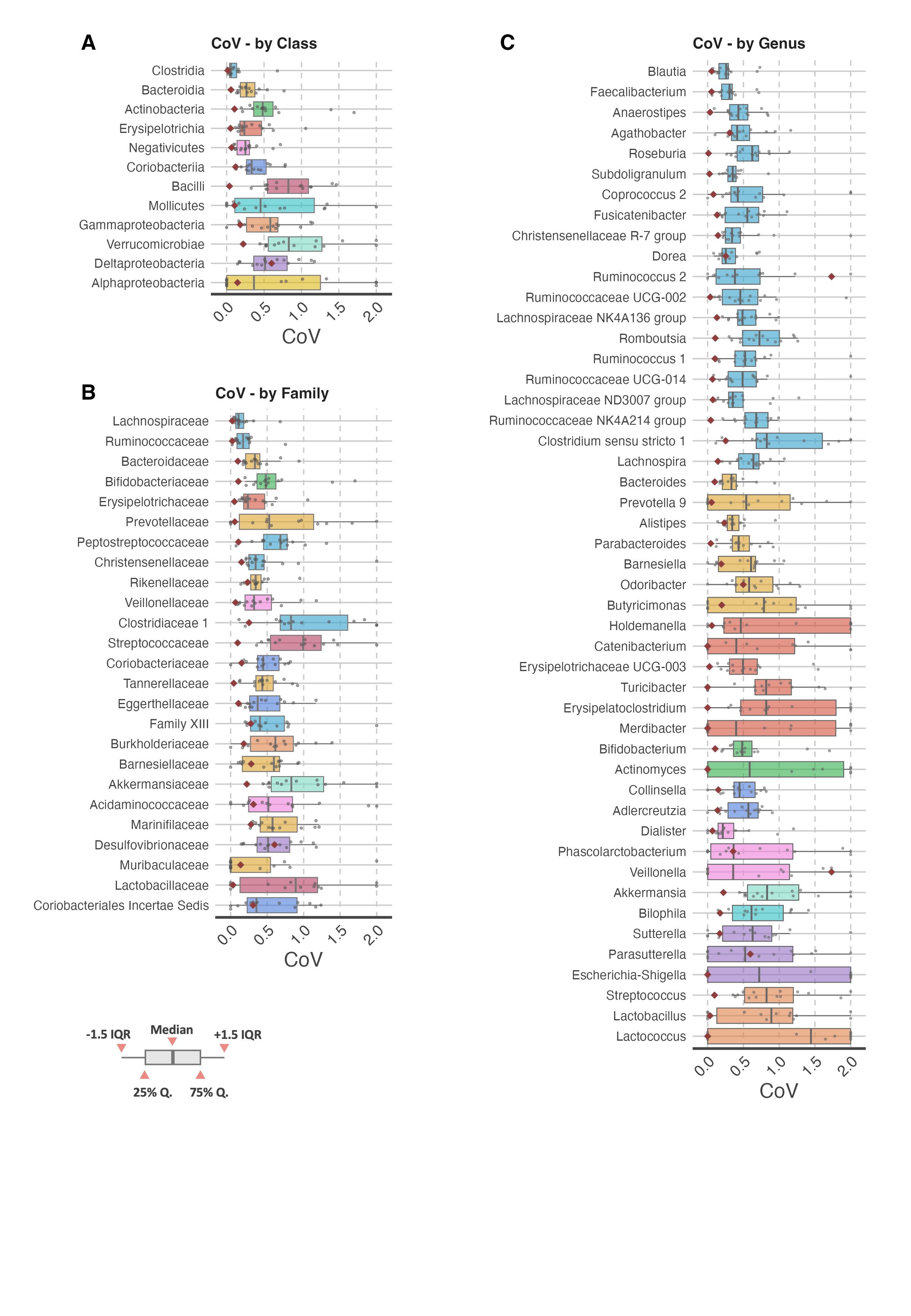

Supplement: Supplemental Material [file KGMI_A_2427878_SM0472.zip › KGMI 2427878 updated/kgmi-s-2024-0526-20241106211645/Suppl_Figure_2.tiff]

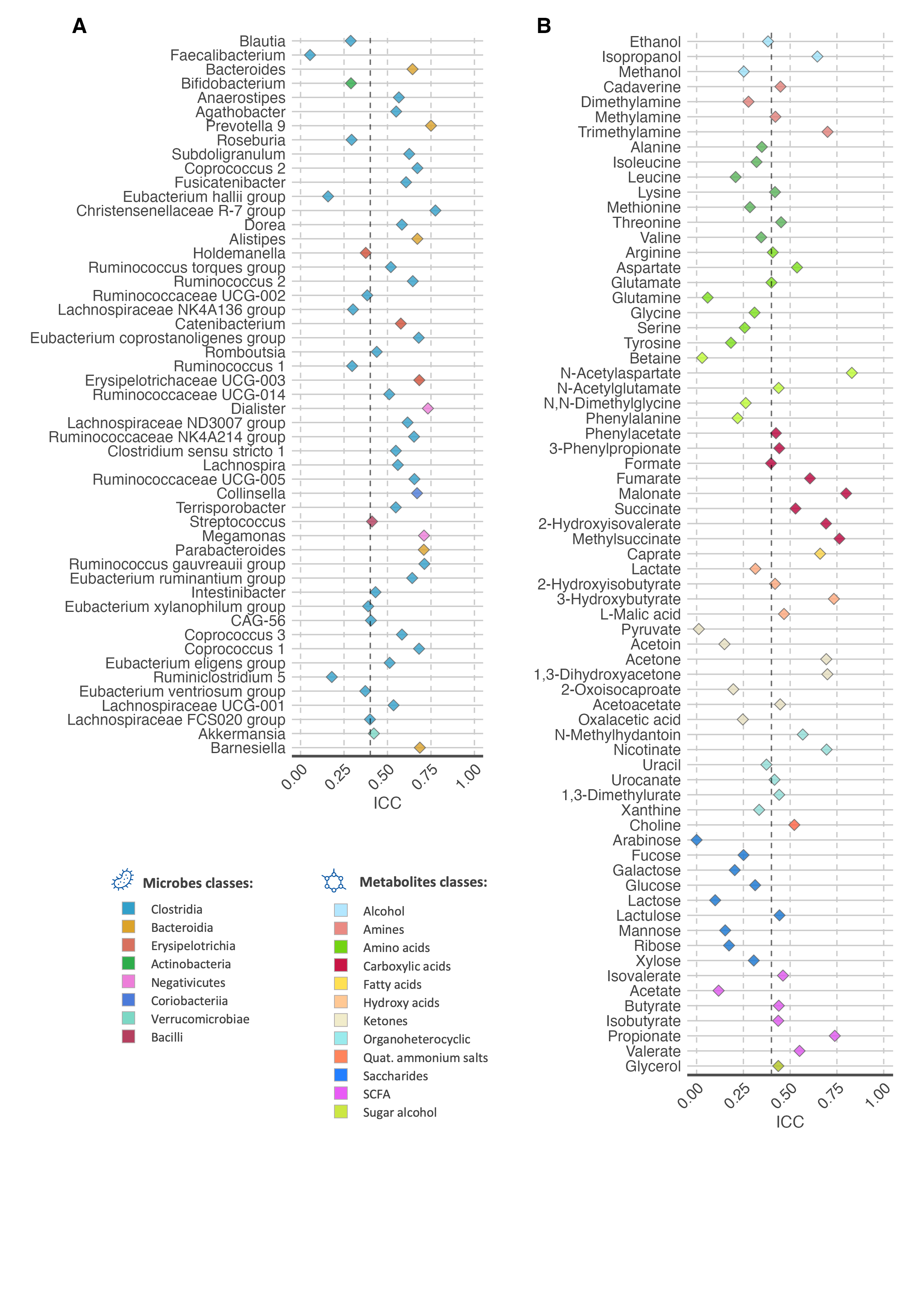

Supplement: Supplemental Material [file KGMI_A_2427878_SM0472.zip › KGMI 2427878 updated/kgmi-s-2024-0526-20241106211645/Suppl_Figure_3.tiff]

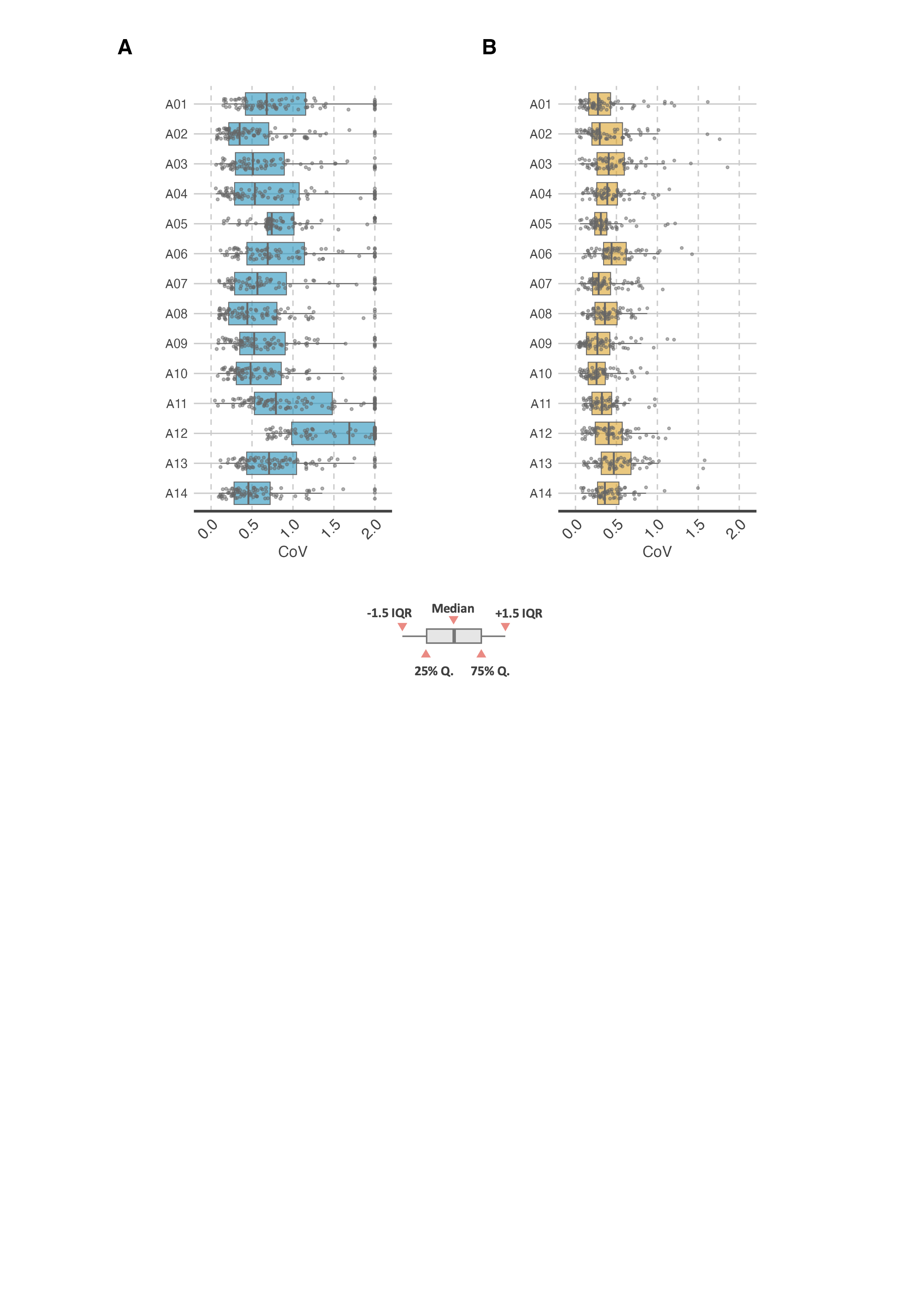

Supplement: Supplemental Material [file KGMI_A_2427878_SM0472.zip › KGMI 2427878 updated/kgmi-s-2024-0526-20241106211645/Suppl_Figure_4.tiff]

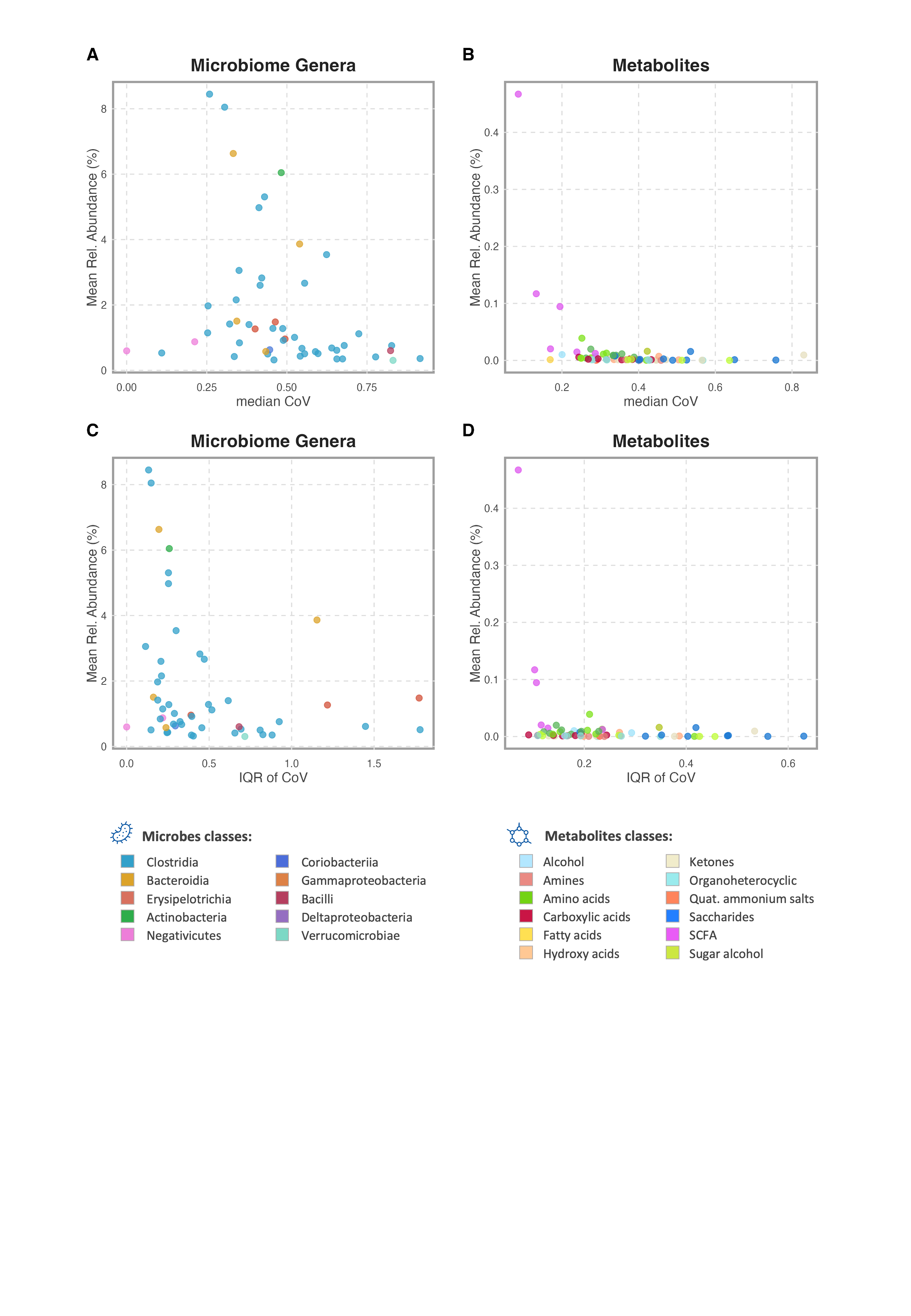

Supplement: Supplemental Material [file KGMI_A_2427878_SM0472.zip › KGMI 2427878 updated/kgmi-s-2024-0526-20241106211645/Suppl_Figure_5.tiff]

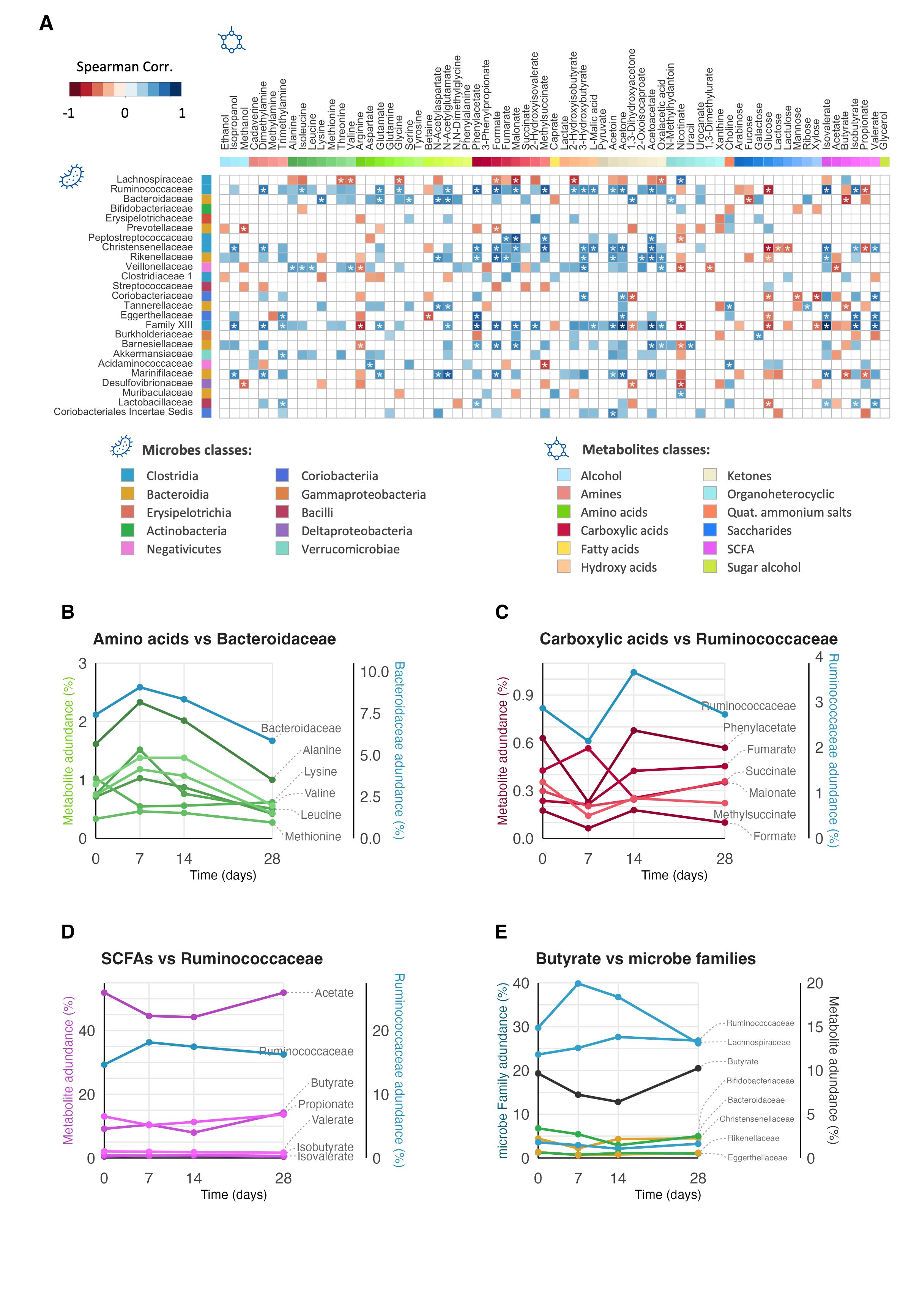

Supplement: Supplemental Material [file KGMI_A_2427878_SM0472.zip › KGMI 2427878 updated/kgmi-s-2024-0526-20241106211645/Suppl_Figure_6.tiff]
